# Supplementary material for: Light activation of Orange Carotenoid Protein reveals bicycle-pedal single-bond isomerization
Source: Nat Commun. 2022 Oct 28;13:6420. doi: 10.1038/s41467-022-34137-4 (PMC9616832; doi:10.1038/s41467-022-34137-4)
Supplement: Supplementary file 1 — Supplementary Information [file 41467_2022_34137_MOESM1_ESM.pdf]

Supplementary information

Light activation of Orange Carotenoid Protein reveals bicycle-pedal single-bond isomerization

V.U. Chukhutsina*, J. M. Baxter*, Alisia Fadini*, R.M. Morgan*, M.A. Pope*, K. Maghlaoui*, C.M. Orr#, A.Wagner#, J.J. van Thor*

*Department of Life Sciences, Imperial College London, London SW7 2AZ, United Kingdom
#Diamond Light Source, United Kingdom

Supplementary note 1: Monitoring the OCP photocycle and primary photoproduct in solution by UV-VIS spectroscopy

To confirm that the C9'-C8' *cis* and C7'-C6' *trans* (bicycle-pedal isomer) of CAN in I1 thermally relaxes back to C9'-C8' *trans* and C7'-C6' *cis* after 2 min of illumination and rule out a light-driven process, we collected time-dependent UV-VIS spectra upon 40 s of illumination followed by 5 min of darkness (Supplementary Fig 1 a). Similar to the experiment from Fig 8a, all the spectra collected within 5 min 40 s were fit simultaneously using global fit approach[1–3] . In these experimental conditions relaxation of the bicycle-pedal CAN isomer, if light driven, should not exhibit a spectral characteristic of EAS3 observed in Fig 8a upon continuous illumination.

The results of the global fitting of the dataset highlight four EAS components (Supplementary Fig 1 a). The EAS2 component from Supplementary Fig 1 a (red solid line), like its counterpart in Fig 8a, due the spectral similarity of the two spectra, can with confidence be assigned to the formation of the bicycle-pedal isomer like in Fig 8a. The EAS3 component (Supplementary Fig 1 a, dotted line) shows a 25 nm red-shifted absorption maximum similar to that observed in Fig. 8a, thereby confirming that the bicycle-pedal isomer thermally relaxes to its “dark” C9'-C8' *trans* and C7'-C6' *cis* conformation in 2 min to yield a new OCP^R-like intermediate. The steady-state EAS4 component represents recovery of the OCP^R-like intermediate state to OCP^O, similar to the OCP^R-OCP^O conversion occurring in darkness.

To demonstrate the light intensity dependence of both the rate of accumulation of the primary product and photocycle intermediates, we monitored absorption changes of OCP at different power densities and two characteristic wavelengths: 470 nm (OCP^O/Isom) and 550 nm (OCP^R-like, OCP^R) (Supplementary Fig 1 b-c). The results show that the rates of absorption changes collected at both wavelengths are proportional to illumination intensities.

Supplementary note 2: Light-driven salt bridge formation and Cl^- displacement in OCP

As can be seen in Fig 6b, the position of R155 is modulated by HOH41 and HOH46 via their H-bonding with R155. In I1, the strength of HOH41-R155 and HOH46-R155 H-bonds increases as their hydrogen donor – hydrogen acceptor distances decrease by 0.3 Å and 0.8 Å respectively. This H-bond rearrangement is controlled by the chloride ion which is located close to the domain interface in the CTD where it coordinates four water molecules (HOH13, HOH46, HOH41 and HOH23) within a 3.0-4.0 Å radius through ion-dipole interactions (Supplementary Fig 2 a).

In the I1-I3 intermediates, a small displacement of the chloride ion by 0.2 Å coincides with small displacements of all four water molecules it is hydrogen-bonded to through ion-dipole forces (Supplementary Fig 2 a, Fig 6b).
After 2-5 min of illumination (I2, I3, Fig 6b), the HOH46-R155 distance remains the same as in I1 whereas the HOH41-R155 distance reverts back to its original value in OCP^O. After 10 min the HOH46-R155 hydrogen bond strength turns from moderate to strong since its distance decreases down to 2.5 Å. Unlike water molecules, all CTD amino acids in I1 located within a 4 Å radius from Cl^- (W277, P276, T275 and L248) remain in their position upon illumination, with the exception of a marginal (<<0.2 Å) displacement of P276.

In I2-I3 these amino-acids are displaced by 0.2 Å, coinciding with the movement of other CTD amino acids located at the domain interface. In I4 all amino acids within 4 Å from Cl^- undergo a translocation by 0.5 Å with Cl^- moving in the same direction. Overall, the movement of the chloride ion observed in the first 5 min of illumination is mainly caused by the H-bond rearrangement in the water channel (Cl1, Fig 7, Supplementary Fig 2 a), while in I4 it is mainly caused by translocation of the CTD away from NTD (Supplementary Fig 2 b).

Supplementary note 3: water molecules in FTIR data

It should be kept in mind that the positive and negative features of the EAS FTIR spectra (Fig 8c,d and Supplementary Fig 1 f) are the result of broad overlapping contributions from positive and negative spectral signatures as well as one or more vibration modes giving a signal at the same wavenumber. Since it is not possible to disentangle such overlapping contributions, it is difficult to assign spectral features solely to a single vibration process.

The most complicated spectral features are in the 1600−1645 cm^{-1} region, where the spectral differences between the H_2O and the D_2O spectra are not easily interpretable by simple H/D substitution. For instance, the ~1600−1645 cm^{-1} band (in H_2O) observed between 40-120 s after the illumination onset (EAS2-3, Supplementary Fig 1 f), completely disappears in the presence of D_2O (Fig 8c). Similar FTIR signature has been observed previously in OCP where it was tentatively assigned to H_2O vibration modes[4]. Indeed, only the HOH symmetric mode vibration (1643 cm^{-1}) contributes in the observed region (1700 cm^{-1}-1500 cm^{-1}) whereas the DOD symmetric vibration band lies at 1210 cm^{-1}, (Supplementary Fig 1 g). This therefore suggests that the ~1600−1645 cm^{-1} shoulder could be assigned to vibrational motions of HOH. In support of this assignment, we observed that the presence of the 1600-1640 cm^{-1} band in the H_2O data correlates with the light-driven displacement of water molecules observed in the crystallographic data on the same time scale (Fig 7- I2 and I3). The rearrangement of water molecules affects the two water clusters CL1 and CL2 each centred on HOH46 and HOH152 respectively. The strongest DED signals are observed in CL1, which is located on the interface between the two domains (Fig 7, Supplementary Fig 1 f).

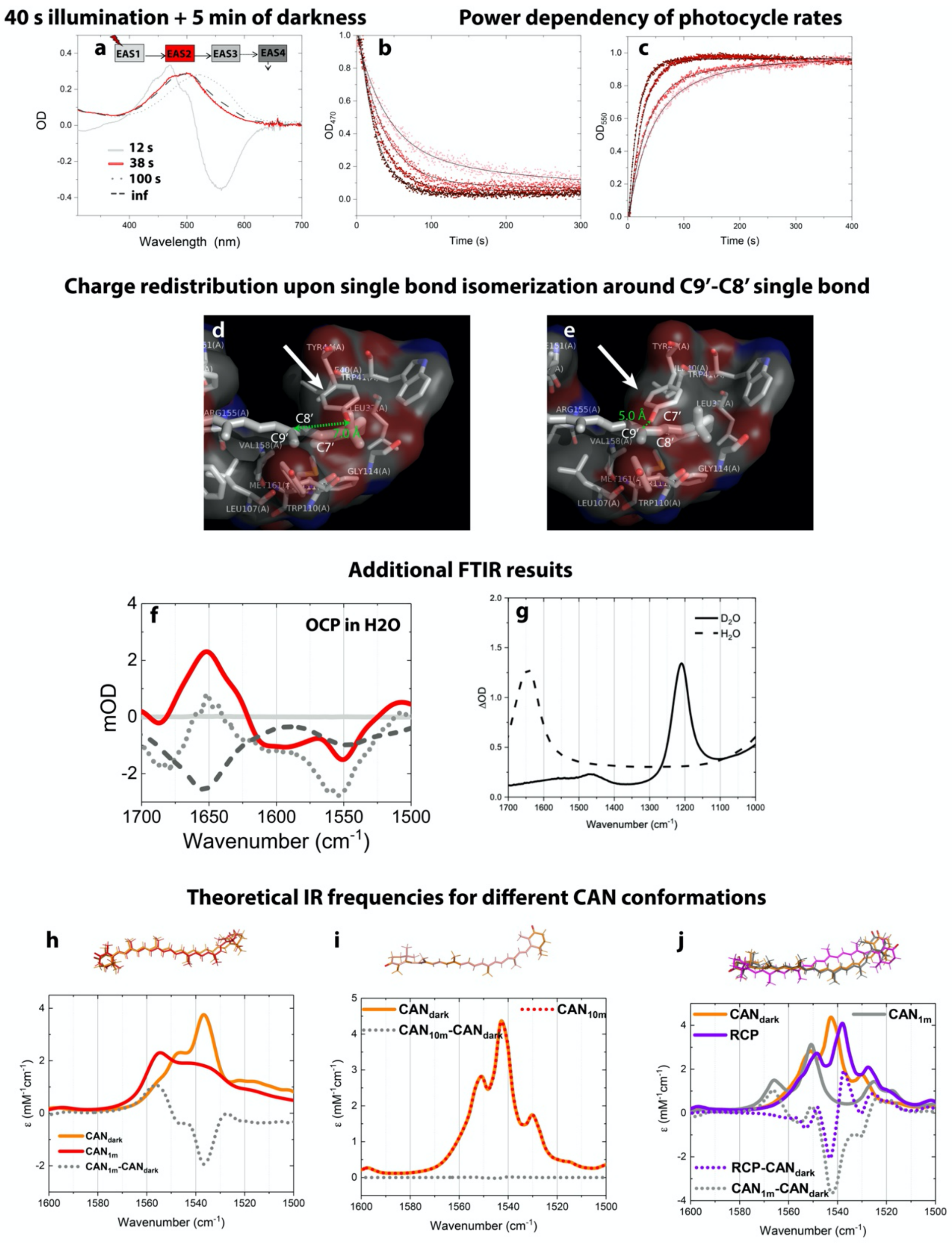

Supplementary Fig. 1 Spectroscopic changes in OCP upon illumination (a) Results of the global fit of UV-VIS absorption kinetics in solution upon 40 s of illumination followed by 5 min of darkness. The kinetic model used for the fit is indicated at the top of the graph which also shows the lifetimes. (b and c) Power dependency profile of photocycle intermediate accumulation rates upon continuous illumination with violet light (410 nm) as monitored by 470 nm bleach (b) and 550 nm rise (c). Power densities used going from dark red to pink: 3.5 mW cm^{-2}, 2.8 mW cm^{-2}, 1.0 mW cm^{-2} and 0.5 mW cm^{-2}. Black lines represent double exponential fits of the curves. (d-e) Zoomed view of the carotenoid tunnel near the β2 ring in OCP^{O} (d) and I1 (e) states. The two single bonds isomerization (C9'-C8' and C7'-C6') in a bicycle pedal manner causes charge redistribution in the C9'-C8'=C7'-C6' CAN moiety. (f) Global analysis results obtained for OCP FTIR signals (H_2O) from the simultaneous global fit

of UV-VIS/FTIR data. (g) FTIR spectra of H_2O (dashed line) and D_2O (solid line) alone. (h-j)Theoretical IR intensities were calculated using Harmonic frequency calculations (HFC) at the B3LYP/6-311+G level. (h) IR values were calculated for CAN in the two states CAN_{Dark} and CAN_{I1} when dihedral angles were freed in the calculation of the optimised geometry. (i) Calculated IR spectra for CAN_{dark} and CAN_{10m}. (j) Calculated IR spectra for CAN_{dark}, CAN_{1m} and RCP. Source data are provided as a Source Data file.

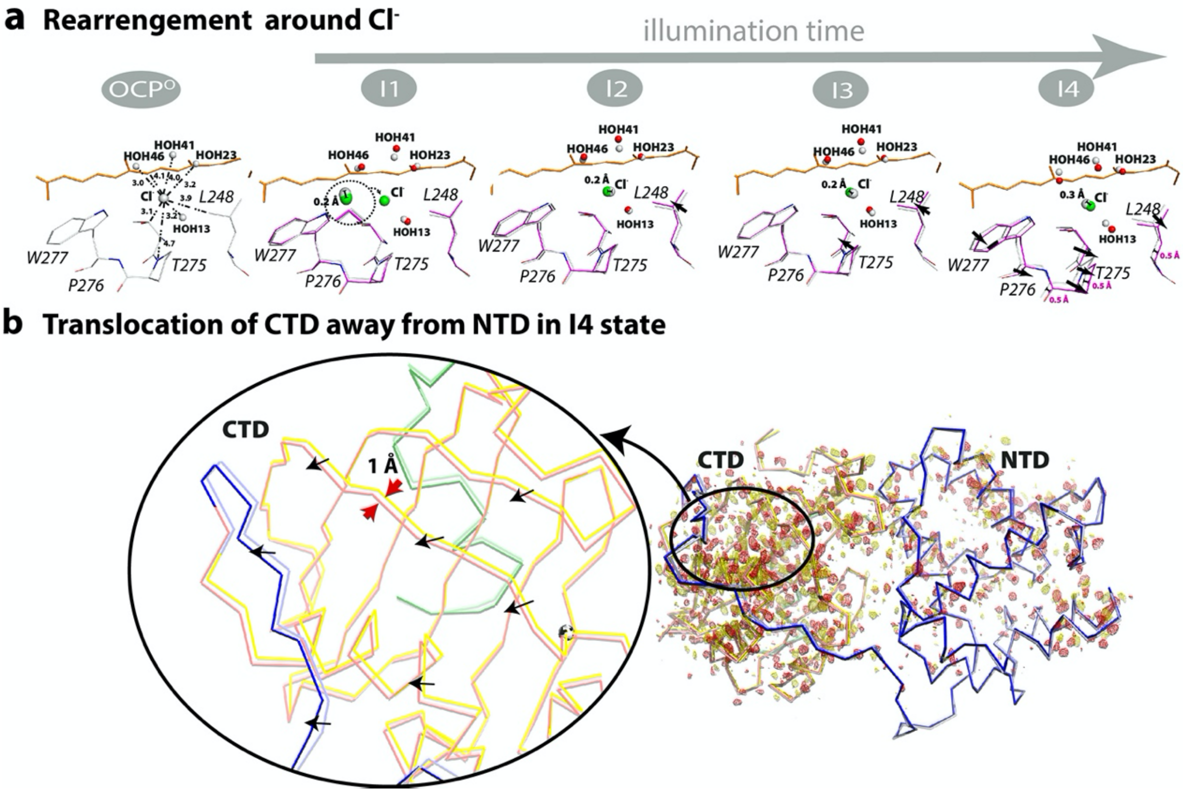

Supplementary Fig. 2 Structural changes in OCP upon illumination (a) Light-driven structural changes in I1-I4 states. Features shown are within a 4 Å radius from the Cl^- ion. The blue and red mesh represent DED maps contoured at ±3.0σ. Dark state amino acids, water and Cl^- coordinates are shown in grey while light state extrapolated coordinates are shown in pink sticks, red (HOH) and green (Cl^-) spheres, respectively. (b) Translocation of CTD from NTD in I4. DED maps obtained for F_{I4}–F_{Dark} (10 min) contoured at ±3.0σ (yellow and red for the appearance and disappearance of density respectively) and extrapolated coordinates for I4 vs OCP^O showing a 1 Å translocation of CTD away from NTD.

Supplementary Table 1: X-ray crystallography data collection and refinement statistics of OCP in the dark and four illuminated states I1-I4.

state	OCP^O			OCP^O			OCP^O		
dataset	1			2			3		
	**Overall**	**InnerShell**	**OuterShell**	**Overall**	**InnerShell**	**OuterShell**	**Overall**	**InnerShell**	**OuterShell**
Low resolution limit	71.48	71.48	1.46	30	30	1.35	87.29	87.29	1.4
High resolution limit	1.42	6.35	1.42	1.32	5.9	1.32	1.36	6.08	1.36
Rmerge (within I+/I-)	0.085	0.051	2.65	0.073	0.051	2.062	0.075	0.043	3.543
Rmerge (all I+ and I-)	0.086	0.052	2.735	0.074	0.053	2.15	0.076	0.043	3.624
Rmeas (within I+ and I+/I-)	0.089	0.053	2.805	0.076	0.054	2.239	0.079	0.045	3.73
Rmeas (all I+ & I-)	0.089	0.053	2.813	0.076	0.055	2.239	0.078	0.044	3.718
Rpim (within I+/I-)	0.027	0.016	0.915	0.024	0.017	0.864	0.025	0.014	1.165
Rpim (all I+ & I-)	0.02	0.012	0.656	0.017	0.013	0.618	0.018	0.01	0.83
Rmerge in top intensity bin	0.051	-	-	0.049	-	-	0.039	-	-
Total number of observations	1288728	14702	86669	1510774	15713	76625	1465081	17776	108032
Total number unique	65306	824	4777	81068	1001	5938	74252	939	5438
Mean((I)/sd(I))	15.3	50	1.1	17.1	48	1.2	17.1	63.4	1.1
Mn(I) half-set correlation CC(1/2)	0.999	0.999	0.526	0.999	0.999	0.535	1	0.999	0.53
Completeness	100	99.9	100	100	98.5	100	100	100	100
Multiplicity	19.7	17.8	18.1	18.6	15.7	12.9	19.7	18.9	19.9
Mean(Chi^2)	0.93	0.82	0.89	0.94	0.87	0.91	0.94	1.03	0.88
Anomalous completeness	100	99.6	100	100	96.8	100	100	100	100
Anomalous multiplicity	10.2	10.4	9.2	9.5	9	6.4	10.2	11	10.1
DelAnom correlation between half-set	-0.013	0.12	0.03	0.129	0.489	-0.007	-0.252	-0.36	-0.003
Mid-Slope of Anom Normal Probability	0.932	-	-	0.941	-	-	0.826	-	-
**Average unit cell**	82.54 82.54 87.35 90 90 120			82.54 82.54 87.36 90 90 120			82.59 82.59 87.29 90 90 120		
**Space group**	$P3_221$			$P3_221$			$P3_221$		

state	I1			I1			I2		
dataset	1			2			1		
	**Overall**	**InnerShell**	**OuterShell**	**Overall**	**InnerShell**	**OuterShell**	**Overall**	**InnerShell**	**OuterShell**
Low resolution limit	71.49	71.49	1.35	37.39	37.39	1.5	29.07	29.07	1.46
High resolution limit	1.32	5.9	1.32	1.46	6.53	1.46	1.42	6.35	1.42
Rmerge (within I+/I-)	0.049	0.026	2.448	0.066	0.043	2.979	0.065	0.028	2.708
Rmerge (all I+ and I-)	0.05	0.027	2.528	0.067	0.044	3.104	0.067	0.03	2.79
Rmeas (within I+ and I+/I-)	0.051	0.028	2.661	0.069	0.046	3.162	0.069	0.03	2.867
Rmeas (all I+ & I-)	0.051	0.028	2.634	0.069	0.045	3.197	0.068	0.031	2.871
Rpim (within I+/I-)	0.016	0.009	1.02	0.022	0.014	1.054	0.021	0.01	0.94
Rpim (all I+ & I-)	0.012	0.007	0.721	0.016	0.011	0.762	0.015	0.008	0.673
Rmerge in top intensity bin	0.024	-	-	0.043	-	-	0.032	-	-
Total number of observations	1514258	18368	76396	1066826	13743	77009	1271095	12807	86302
Total number unique	80954	1015	5917	60537	768	4419	65092	806	4775
Mean((I)/sd(I))	23.8	95.8	1.2	19.1	57.3	1	19.1	67.3	1.1
Mn(I) half-set correlation CC(1/2)	1	0.999	0.509	0.999	0.999	0.448	1	0.999	0.52
Completeness	100	99.7	100	100	99.4	100	100	98.5	100
Multiplicity	18.7	18.1	12.9	17.6	17.9	17.4	19.5	15.9	18.1
Mean(Chi^2)	0.94	1.04	0.86	0.98	0.93	0.87	0.94	0.64	0.95
Anomalous completeness	100	99.5	99.9	99.8	99.9	100	100	98.5	100
Anomalous multiplicity	9.6	10.5	6.4	9	10.6	8.8	10	9	9.1
DelAnom correlation between half-set	-0.103	0.266	-0.025	-0.074	-0.058	-0.048	0.131	0.728	-0.025
Mid-Slope of Anom Normal Probability	0.876	-	-	0.98	-	-	0.912	-	-
**Average unit cell**	82.55 82.55 87.21 90 90 120			82.68 82.68 87.66 90 90 120			82.48 82.48 87.21 90 90 120		
**Space group**	$P3_221$			$P3_221$			$P3_221$		

state	I2			I3			I3		
dataset	2			1			2		
	**Overall**	**InnerShell**	**OuterShell**	**Overall**	**InnerShell**	**OuterShell**	**Overall**	**InnerShell**	**OuterShell**
Low resolution limit	55.29	55.29	1.37	55.5	55.5	1.44	87.07	87.07	1.32
High resolution limit	1.34	5.99	1.34	1.4	6.26	1.4	1.29	5.77	1.29
Rmerge (within I+/I-)	0.053	0.027	2.763	0.133	0.054	10.905	0.043	0.028	1.63
Rmerge (all I+ and I-)	0.054	0.028	2.847	0.135	0.055	11.077	0.044	0.029	1.725
Rmeas (within I+ and I+/I-)	0.056	0.029	2.971	0.14	0.057	11.51	0.045	0.03	1.82
Rmeas (all I+ & I-)	0.055	0.029	2.951	0.138	0.057	11.372	0.045	0.03	1.824
Rpim (within I+/I-)	0.017	0.009	1.079	0.044	0.018	3.645	0.014	0.009	0.813
Rpim (all I+ & I-)	0.013	0.007	0.767	0.031	0.013	2.554	0.01	0.007	0.583
Rmerge in top intensity bin	0.024	-	-	0.05	-	-	0.025	-	-
Total number of observations	1474726	17861	81503	1364917	16392	100741	1564913	19356	60623
Total number unique	77412	973	5680	68765	865	5049	86400	1081	6302
Mean((I)/sd(I))	23	100.1	1	14.4	45.5	1.4	25.6	95.6	1.2
Mn(I) half-set correlation CC(1/2)	1	1	0.468	0.998	0.985	0.739	1	0.999	0.561
Completeness	100	99.8	100	100	99.6	100	100	99.4	100
Multiplicity	19.1	18.4	14.3	19.8	19	20	18.1	17.9	9.6
Mean(Chi^2)	0.91	1.1	0.84	0.97	0.92	1.01	0.89	1.07	0.74
Anomalous completeness	100	99.9	100	99.9	99.7	99.7	100	99.3	99.6
Anomalous multiplicity	9.8	10.8	7.3	10.2	11.1	10.1	9.3	10.3	4.8
DelAnom correlation between half-set	-0.034	0.067	-0.029	-0.154	-0.243	0.004	-0.118	-0.127	0.019
Mid-Slope of Anom Normal Probability	0.899	-	-	0.797	-	-	0.893	-	-
**Average unit cell**	82.52 82.52 87.26 90 90 120			82.81 82.81 87.66 90 90 120			82.50 82.50 87.07 90 90 120		
**Space group**	$P3_221$			$P3_221$			$P3_221$		

state	I4			I4		
dataset	1			2		
	**Overall**	**InnerShell**	**OuterShell**	**Overall**	**InnerShell**	**OuterShell**
Low resolution limit	87.01	87.01	1.5	29.86	29.86	1.41
High resolution limit	1.46	6.53	1.46	1.37	6.13	1.37
Rmerge (within I+/I-)	0.092	0.047	4.332	0.046	0.026	2.561
Rmerge (all I+ and I-)	0.093	0.047	4.419	0.046	0.027	2.654
Rmeas (within I+ and I+/I-)	0.096	0.049	4.564	0.048	0.027	2.74
Rmeas (all I+ & I-)	0.095	0.048	4.536	0.048	0.028	2.745
Rpim (within I+/I-)	0.03	0.015	1.43	0.015	0.009	0.966
Rpim (all I+ & I-)	0.021	0.011	1.017	0.011	0.007	0.692
Rmerge in top intensity bin	0.045	-	-	0.024	-	
Total number of observations	1182100	14432	87696	1378407	14775	80781
Total number unique	59958	773	4396	71802	900	5231
Mean((I)/sd(I))	16.5	53.3	1.4	24.6	94	1
Mn(I) half-set correlation CC(1/2)	0.999	0.999	0.688	1	1	0.533
Completeness	100	100	100	100	98.6	100
Multiplicity	19.7	18.7	19.9	19.2	16.4	15.4
Mean(Chi^2)	0.95	1.01	0.83	0.88	0.88	0.76
Anomalous completeness	100	100	99.9	100	98.6	100
Anomalous multiplicity	10.2	11	10.2	9.9	9.5	7.8
DelAnom correlation between half-set	-0.323	-0.47	-0.005	-0.104	0.275	-0.006
Mid-Slope of Anom Normal Probability	0.782	-	-	0.882	-	-
**Average unit cell**	82.58 82.58 87.01 90.00 90.00 120.00			82.44 82.44 86.63 90.00 90.00 120.00		
**Space group**	$P3_221$			$P3_221$		

**Refinement**	OCP^O 1	OCP^O 2	OCP^O 3	I1 1	I1 2	I2 1	I2 2	I3 1	I3 2	I4 1	I4 2
Resolution	55.39-1.43	30.02-1.32	55.40-1.36	55.35-1.32	37.42-1.46	29.09-1.42	55.35-1.34	55.36-1.39	71.55-1.29	71.62-1.46	29.89-1.37
No. reflections all/free	63888 / 3134	81019 / 3975	74207 / 3699	80887 / 3975	60499 / 2818	65042 / 3188	77359 / 3895	69455 / 3445	86348 / 4173	59930 / 2794	71743 / 3474
R-factor/R-free	0.168 / 0.203	0.170 / 0.192	0.172 / 0.196	0.167 / 0.192	0.164 / 0.192	0.168 / 0.200	0.170 / 0.192	0.1656 / 0.189	0.171 / 0.1954	0.163 / 0.190	0.175 / 0.217
RMS Deviations											
Bonds	0.0121	0.0148	0.0142	0.015	0.0132	0.0131	0.0147	0.01485	0.0149	0.0133	0.0133
Angles	1.871	2.032	1.988	2.054	1.893	1.91	2.012	2.015	2.028	1.98	1.946
Chain mean B											
(No. atoms)											
AAA	25.5(5563)	23.5(5671)	24.4(5563)	24.0(5668)	29.7(5626)	26.4(5563)	24.8(5563)	27.6(5563)	23.7(5587)	26.7(5680)	27.2(5671)
AaA	18.8(94)	16.6(94)	17.5(94)	17.8(94)	22.9(94)	19.5(94)	17.9(94)	20.9(94)	17.3(94)	19.7(94)	19.6(94)
AbA	27.0(12)	25.1(12)	26.9(12)	26.6(12)	32.8(12)	28.5(12)	26.7(12)	30.0(12)	25.3(12)	29.8(12)	29.0(12)
AcA	70.5(7)	72.0(7)	65.7(7)	78.2(7)	82.0(7)	72.1(7)	68.3(7)	69.3(7)	64.2(7)	72.5(7)	68.6(7)
BBB	41.5(449)	39.7(437)	41.9(449)	41.7(494)	46.5(449)	42.5(449)	41.3(49)	44.4(449)	40.4(471)	45.7(543)	45.4(512)
CCC	20.9(1)	18.7(1)	20.1(1)	19.7(1)	25.1(1)	21.3(1)	19.9(1)	22.9(1)	19.4(1)	22.1(1)	22.9(1)
**Ramachandran**											
In preferred regions	192 (94.58%)	191 (95.5%)	193 (95.07%)	196 (96.17%)	192 (95.52%)	192 (94.58%)	193 (95.57%)	194 (95.57%)	194 (95.57%)	186 (93.47%)	188 (94.0%)
in allowed regions	11 (5.42%)	9 (4.5%)	10 (4.93%)	7(3.83%)	9(4.48%)	11 (5.42%)	9 (4.43%)	9 (4.43%)	9 (4.43%)	13(6.53%)	12(6.0%)
Outliers	0 (0%)	0 (0%)	0 (0%)	0 (0%)	0 (0%)	0 (0%)	0 (0%)	0 (0%)	0 (0%)	0 (0%)	0 (0%)

Supplementary Table 2: Cl^- and water molecules located within 6 Å of the carotenoid and in the newly identified water cluster (CL2). The molecules that are either conserved or found at the same location (within 0.5 Å) as in previously reported OCP structures (PDB entries 4XB5 [http://doi.org/10.2210/pdb4xb5/pdb], 5TUX [http://doi.org/10.2210/pdb5tux/pdb], 5UI2 [http://doi.org/10.2210/pdb5UI2/pdb]) are indicated.

Name in PDB (current study)	B Factor	Nearest residue(s) that can form H bond	Distance, Å	Corresponding molecule in 4XB5	Corresponding molecule in 5TUX	Corresponding molecule in 5UI2	Location and associated water cluster (CL1 or CL2 as in Fig 7)
HOH2	25.49 (A)/28.03 (B)	HOH217/O (A)/HOH93/O (B)	1.6(A)/1.7 (B)	HOH743	HOH756	HOH660	major interface/CL1
HOH23	23.26	HOH217/O and HOH385/O	2.1	HOH693	HOH602	HOH586	major interface/CL1
HOH30	25.77	TYR44/OH	2.3	HOH691	HOH505	No	TYR44
HOH46	27.35	ASN104/OD1	2.1	HOH699	HOH561	HOH561	major interface/CL1
HOH53	25.85	HOH256/O	2.5	No	No	No	between GLN36 and MET161
HOH57	23.43	No	No	HOH727	HOH577	HOH573	major interface/ CL1
HOH93	29.88	HOH2/O (B)	2.1	HOH728	HOH758	HOH661	major interface/CL1
HOH153	49.19	HOH421	1.9	HOH819	HOH521	No	major interface/CL1
HOH217	39.21	HOH2/O(A)	1.7	No	No	HOH647	major interface/CL1
HOH256	31.53	HOH53/O	2.2	No	No	No	CAN/O (keto group)
HOH320	64.68	No	No	No	No	No	MET47
HOH385	39.09	HOH2/O(A) and HOH424/O	1.8	HOH719	HOH720	No	major interface/ CL1
HOH424	67.81	HOH93/O	1.4	No	No	No	major interface/ CL1
HOH132	36.53	No	No	No	No	No	CL2, GLU34
HOH229	45.03	LEU77/O	2.7	No	No	No	CL2, LEU77
HOH250	42.7	HOH401/O	2.1	No	No	No	CL2, GLU250
HOH401	49.9	HOH250/O	1.9	No	No	No	CL2, THR80
HOH420	56.95	HOH229/O	3	No	No	No	CL2, THR80
Cl	20.16	-	-	HOH673	HOH699	Cl	

Supplementary Table 3: X-ray crystallography data collection, refinement statistics and anomalous peaks for OCP for long-wavelength experiment. Statistics for the highest-resolution shell are shown in parentheses.

**Energy**	**2.9 keV**	**2.8 keV**
**Wavelength (Å)**	4.28	4.43
**Resolution range**	72 – 2.8 (2.95 – 2.8)	72 – 2.89 (3.07 – 2.89)
**Space group**	$P3_221$	$P3_221$
**Unit cell (Å, °)**	82.67 82.67 87.58	82.660 82.660 87.580
	90 90 120	90 90 120
**Total reflections**	190330 (15166)	171371 (14952)
**Unique reflections**	8674 (1188)	7891 (1194)
**Multiplicity**	21.9 (12.8)	21.7 (12.5)
**Completeness (%)**	98.3 (94.5)	98.0 (93.6)
**Mean I/sigma(I)**	18.9 (6.8)	14.6 (4.8)
**Wilson B-factor**	31.5	32.5
**R-merge**	0.138 (0.304)	0.173 (0.413)
**R-meas**	0.140 (0.316)	0.177 (0.429)
**R-pim**	0.027 (0.082)	0.035 (0.111)
**CC1/2**	0.998 (0.976)	0.997 (0.963)

Anomalous peaks

**2.9 keV**			**2.8 keV**		
**Peak Height (σ)**	**Distance (Å)**	**Nearest Residue**	**Peak Height (σ)**	**Peak Height (σ)**	**Peak Height (σ)**
14.43	0.174	CYS245	12.9	12.9	12.9
14.18	0.14	MET202	12.6	12.6	12.6
14.12	0.502	MET161	12.07	12.07	12.07
13.98	0.185	MET284	11.81	11.81	11.81
**13.93**	**0.452**	**CL1**	11.58	11.58	11.58
13.35	0.144	MET74	11.54	11.54	11.54
12.69	0.212	CYS95	11.32	11.32	11.32
11.56	0.167	MET83	10.73	10.73	10.73
11.32	0.152	MET117	9.32	9.32	9.32
10.83	0.346	CYS84	7.7	7.7	7.7
**8.52**	**3.375**	**ASN285**	6.01	6.01	6.01
7.42	0.104	MET47			
7.1	0.174	MET61			
**7.06**	**0.802**	**ASN14**			

*Cl anomalous peaks highlighted in green.

Supplementary Table 4: Light-driven newly formed H-bonds in the NTD main chain. The H-bond list was obtained using the list_mc_hbonds.py script from Robert L. Campbell (http://pldserver1.biochem.queensu.ca/~rlc/work/pymol/). Water molecules were excluded from the calculations.

		Distances, Å			
H-bond (inter subunit only)		I1	I2	I3	I4
A/ILE`25/N	A/PRO`22/O	3.45	3.39		
A/ARG`27/N	A/THR`24/O	3.53	3.38	3.26	
A/LEU`37/N	A/GLU`34/O	3.35	3.32		3.29
A/ALA`38/N	A/ASP`35/O	3.35	3.34		
A/THR`52/N	A/THR`52/O	3.09			
A/SER`60/N	A/GLY`57/O	3.05	3.2	3.14	
A/GLU`65/N	A/LEU`63/O	3.66	3.61		
A/LYS`69/N	A/ASN`66/O	3.72	3.59	3.33	3.36
A/CYS`84/N	A/GLN`81/O	3.31	3.27		3.22
A/ALA`87/N	A/CYS`84/O	3.00	3.09	3.28	
A/TYR`98/N	A/CYS`95/O	3.39		3.51	3.55
A/LYS`106/N	A/PRO`103/O	3.18	3.32		
A/TYR`111/N	A/GLY`108/O	3.24	3.28	3.28	3.51
A/LEU`113/N	A/TRP`110/O	3.64	3.45	3.26	3.43
A/ALA`140/N	A/ALA`137/O	3.23	3.33	3.32	3.37

* The pattern of newly formed and disrupted hydrogen bonds between intermediates illustrates the transient nature of the hydrogen bonding and agrees with our FTIR results (Fig 8). Out of 15 hydrogen bonds, most remain in the I1 and I2 structural intermediates, 7 do so in I1-I3 while 4 remain in all intermediates.

Supplementary References

1. Snellenburg, J. J., Laptenok, S. P., Seger, R., Mullen, K. M. & van Stokkum, I. H. M. Glotaran: A Java-Based Graphical User Interface for the R Package TIMP. *J. Stat. Softw.* **49**, 1–22 (2012).
2. van Stokkum, I. H. M., Larsen, D. S. & van Grondelle, R. Global and target analysis of time-resolved spectra. *Biochim. Biophys. Acta-Bioenergetics* **1657**, 82–104 (2004).
3. Holzwarth, A. Data Analysis of Time-Resolved Measurements. in *Biophysical Techniques in Photosynthesis* (eds. Amesz, J. & Hoff, A.) **3**, 75–92 (Springer Netherlands, 1996).
4. Mezzetti, A. *et al.* Two-Step Structural Changes in Orange Carotenoid Protein Photoactivation Revealed by Time-Resolved Fourier Transform Infrared Spectroscopy. *J. Phys. Chem. B* **123**, 3259–3266 (2019).
