# Supplementary material for: Light activation of Orange Carotenoid Protein reveals bicycle-pedal single-bond isomerization
Source: Nat Commun. 2022 Oct 28;13:6420. doi: 10.1038/s41467-022-34137-4 (PMC9616832; doi:10.1038/s41467-022-34137-4)
Supplement: Supplementary file 2 — Reporting Summary [file 41467_2022_34137_MOESM2_ESM.pdf]

nature portfolio

Corresponding author(s): van Thor

Last updated by author(s): Oct 10, 2022

Reporting Summary

Nature Portfolio wishes to improve the reproducibility of the work that we publish. This form provides structure for consistency and transparency in reporting. For further information on Nature Portfolio policies, see our Editorial Policies and the Editorial Policy Checklist.

Statistics

For all statistical analyses, confirm that the following items are present in the figure legend, table legend, main text, or Methods section.

n/a	Confirmed	
☒	☐	The exact sample size (*n*) for each experimental group/condition, given as a discrete number and unit of measurement
☒	☐	A statement on whether measurements were taken from distinct samples or whether the same sample was measured repeatedly
☒	☐	The statistical test(s) used AND whether they are one- or two-sided<br>*Only common tests should be described solely by name; describe more complex techniques in the Methods section.*
☒	☐	A description of all covariates tested
☒	☐	A description of any assumptions or corrections, such as tests of normality and adjustment for multiple comparisons
☒	☐	A full description of the statistical parameters including central tendency (e.g. means) or other basic estimates (e.g. regression coefficient) AND variation (e.g. standard deviation) or associated estimates of uncertainty (e.g. confidence intervals)
☒	☐	For null hypothesis testing, the test statistic (e.g. *F*, *t*, *r*) with confidence intervals, effect sizes, degrees of freedom and *P* value noted<br>*Give P values as exact values whenever suitable.*
☒	☐	For Bayesian analysis, information on the choice of priors and Markov chain Monte Carlo settings
☒	☐	For hierarchical and complex designs, identification of the appropriate level for tests and full reporting of outcomes
☒	☐	Estimates of effect sizes (e.g. Cohen's *d*, Pearson's *r*), indicating how they were calculated

Our web collection on statistics for biologists contains articles on many of the points above.

Software and code

Policy information about availability of computer code

Data collection	Gaussian 06 software package, Diamond i23, i03 and i04 data collection software, Bio-Rad FTS 175C FT-IR spectrophotometer,Agilent 8453 spectrophotometer
Data analysis	REFMAC 5.8.0267, DIALS 3.0.4, MOLREP 2.0.281, Glotaran 1.5.1, COOT 0.9.6, ANODE version 2013/1

For manuscripts utilizing custom algorithms or software that are central to the research but not yet described in published literature, software must be made available to editors and reviewers. We strongly encourage code deposition in a community repository (e.g. GitHub). See the Nature Portfolio guidelines for submitting code & software for further information.

Data

Policy information about availability of data

All manuscripts must include a data availability statement. This statement should provide the following information, where applicable:

- Accession codes, unique identifiers, or web links for publicly available datasets
- A description of any restrictions on data availability
- For clinical datasets or third party data, please ensure that the statement adheres to our policy

Coordinates and structure factors have been deposited in the Protein Data Bank under the following accession codes: 7ZSF [https://doi.org/10.2210/pdb7ZSF/pdb] (OCPO), 7ZSG (I1) [https://doi.org/10.2210/pdb7ZSG/pdb], 7ZSH [https://doi. org/10.2210/pdb7ZSH/pdb] (I2), 7ZSI [https://doi. org/10.2210/pdb7ZSI/pdb] (I3) and 7ZSJ [https://doi.org/10.2210/pdb7ZSJ/pdb] (I4).
Furthermore, the crystal structures of OCP used in this study are available in the Protein Data Bank under the following accession codes: 4XB5 [http:// doi.org/10.2210/pdb4xb5/pdb], 5TUX [http://doi.org/10.2210/pdb5tux/pdb] 5UI2 [http://doi.org/10.2210/pdb5UI2/pdb].
Source data are provided with this paper.

Field-specific reporting

Please select the one below that is the best fit for your research. If you are not sure, read the appropriate sections before making your selection.

☒ Life sciences ☐ Behavioural & social sciences ☐ Ecological, evolutionary & environmental sciences

For a reference copy of the document with all sections, see nature.com/documents/nr-reporting-summary-flat.pdf

Life sciences study design

All studies must disclose on these points even when the disclosure is negative.

Sample size	The sample sizes were not determined.<br>In the X-ray crystallography the beam of X-ray radiation is diffracted from a crystal that contains many copies of the purified protein in a symmetric lattice. Reflections from multiple crystals are detected and recorded, which leads to a large, undetermined, sample size in a diffraction pattern. On top of that, X-ray experiment was repeated at least twice for each time time point on different OCP crystals.<br>Illuminated data at 4 time points were collected, with two replicates for the time points 2 min and 5 min and three replicas for time point 1 min and 10 min. Three replicas for the dark dataset.
Data exclusions	No data exclusions
Replication	At least 2 replicas for each state was collected. All attempts at replication were successful.
Randomization	no randomization. Randomization is not applicable due to the nature of X-ray cryotrapping and spectroscopic solution experiments: illumination for defined period of time should be done.
Blinding	no blinding. The experiments are based on signal detection and automated data analysis and therefore not subject to confirmation bias.

Reporting for specific materials, systems and methods

We require information from authors about some types of materials, experimental systems and methods used in many studies. Here, indicate whether each material, system or method listed is relevant to your study. If you are not sure if a list item applies to your research, read the appropriate section before selecting a response.

Materials & experimental systems

n/a	Involved in the study	
☒	☐	Antibodies
☒	☐	Eukaryotic cell lines
☒	☐	Palaeontology and archaeology
☒	☐	Animals and other organisms
☒	☐	Human research participants
☒	☐	Clinical data
☒	☐	Dual use research of concern

Methods

n/a	Involved in the study	
☒	☐	ChIP-seq
☒	☐	Flow cytometry
☒	☐	MRI-based neuroimaging
